# Invisible Flashes Alter Perceived Sound Location

**DOI:** 10.1038/s41598-018-30773-3

**Published:** 2018-08-17

**Authors:** Patrycja Delong, Máté Aller, Anette S. Giani, Tim Rohe, Verena Conrad, Masataka Watanabe, Uta Noppeney

**Affiliations:** 1 Computational Neuroscience and Cognitive Robotics Centre, University of Birmingham, B15 2TT Birmingham, UK; 2 Max Planck Institute for Biological Cybernetics, 72076 Tübingen, Germany

## Abstract

Information integration across the senses is fundamental for effective interactions with our environment. The extent to which signals from different senses can interact in the absence of awareness is controversial. Combining the spatial ventriloquist illusion and dynamic continuous flash suppression (dCFS), we investigated in a series of two experiments whether visual signals that observers do not consciously perceive can influence spatial perception of sounds. Importantly, dCFS obliterated visual awareness only on a fraction of trials allowing us to compare spatial ventriloquism for physically identical flashes that were judged as visible or invisible. Our results show a stronger ventriloquist effect for visible than invisible flashes. Critically, a robust ventriloquist effect emerged also for invisible flashes even when participants were at chance when locating the flash. Collectively, our findings demonstrate that signals that we are not aware of in one sensory modality can alter spatial perception of signals in another sensory modality.

## Introduction

Information integration across the senses is critical for effective interactions with our natural environment. The extent to which multisensory integration depends on perceptual awareness is controversial^1–4^. According to the global neuronal workspace (GNW) model, consciousness relies on information being broadcast via long-range connectivity in a frontoparietal system^5^. As a result, signals that we are aware of in one sensory modality should be able to influence processing in brain areas dedicated to processing signals from another sensory modality. By contrast, processing of signals that we are not aware of should be largely confined to their own sensory system and have only little effect on perception of signals in another sensory modality.

Indeed, in line with the first prediction, a vast body of research has demonstrated that aware signals from one sensory modality thrust unaware signals in another sensory modality into perceptual awareness according to the classical multisensory principles of temporal coincidence, spatial concordance and semantic and phonological congruency^6–17^. With respect to the spatial ventriloquist illusion, we have recently demonstrated that a sound that we are aware of can boost a flash under dynamic flash suppression into perceptual awareness depending on audiovisual spatial congruency^18^.

By contrast, little evidence has been provided for modulatory effects of unaware signals in one sensory modality on aware signals from another sensory modality. Most notably, the McGurk illusion has been shown to be abolished when visual facial movements are obliterated from awareness under flash suppression^19^ or in bistable perception^20^.

Surprisingly, a recent study demonstrated that participants were faster at responding to supraliminal audiovisually congruent (resp. incongruent) stimuli when those supraliminal stimuli were preceded by subliminal congruent (resp. incongruent) primes^3^. Yet, while these results suggest that the brain can compare auditory and visual letters/phonemes in the absence of awareness, congruency priming does not necessarily imply genuine multisensory interactions. Further, the effects were only observed in terms of response times rather than perceptual representations or choices.

To our knowledge, only one previous study provided tentative evidence that unaware visual signals in patients with hemi-neglect induce a ventriloquist effect and bias patients’ perceived sound location^21^. These results, however, need to be interpreted with caution, as the ventriloquist effect was reported as significant for visual signals only in patients’ neglected, but not in their intact hemifield. Furthermore, this study characterized the ventriloquist effect only for unaware but not for aware visual signals in patients’ neglected hemifield.

In the light of these controversial findings it remains unknown whether unaware signals in one sensory modality can influence conscious perception of signals in another sensory modality. Given accumulating evidence that multisensory interactions emerge already at the primary cortical level^22–27^ one may argue that potentially low-level spatiotemporal information rather than phonological information as in the McGurk illusion may be integrated in the absence of awareness.

Combining the spatial ventriloquist illusion^28,29^ and continuous dynamic flash suppression (dCFS)^30^ we investigated in two psychophysics experiments whether visual signals that observers did not consciously perceive can influence spatial perception of sounds. Critically, we adjusted the saliency of the visual flash, such that the dynamic continuous flash suppression obliterated visual awareness only in a fraction of trials. This allowed us to compare spatial ventriloquism for physically identical flashes that do or do not enter participant’s awareness.

## Methods

### Participants

After giving informed consent, 41 healthy young adults (34 females, 39 right-handed, mean age: 20.1 years, standard deviation: 4.1, range: 18–41) participated in experiment 1, 28 subjects (22 female, 27 right handed, mean age: 19.3 years, standard deviation: 1.4, range: 18–25) in experiment 2. The study was performed in accordance with the principles outlined in the Declaration of Helsinki and was approved by the local ethics review board of the University of Birmingham.

For the first experiment we hypothesized medium effect size (Cohen’s d = 0.5) for the ventriloquist effect in the invisible condition. Hence, we computed sample size (n) for one sided t-test and desired statistical power equal to 0.9, n = 35. To determine sample size for experiment 2 we used effect size based on the sample from the first study (Cohen’s d ≈ 0.7); for the same statistical power (0.9) we obtained n = 18. We continued with data acquisition until the number of included data sets was equal to required sample size (i.e. excluded subjects were replaced; see section exclusion criteria).

### Stimuli and apparatus

Participants sat in a dimly lit room in front of a computer monitor at a viewing distance of 95 cm. They viewed one half of the monitor with each eye using a custom-built mirror stereoscope. Visual stimuli were composed of targets and masks that were presented on a grey, uniform background with a mean luminance of 15.6 cd/m^2^. On the ‘flash present’ trials, one eye viewed the target stimulus (i.e. the flash), which was a grey disc (Ø 0.3°) presented for 50 ms in the upper left, lower left, upper right or lower right quadrant, i.e. at ±3° visual angle along the azimuth and ±1.2° elevation from a grey central fixation dot. The elevation of ±1.2° was selected to enable effective multisensory interactions between flash and sound irrespective of flash elevation. The luminance of the flash was adjusted individually via adaptive staircases to obtain 60% invisible trials. To suppress the flash’s perceptual visibility, four dynamic Mondrians (Ø 2.08°, mean luminance: 48 cd/m^2^) were shown to the other eye^30^. In dynamic CSF original static rectangles^31^ are replaced with dynamically moving gratings^18,30^. The Mondrians were centred on the four potential locations of the target stimuli. Each Mondrian consisted of sinusoidal square gratings (d = 0.6°) which changed their colour and position randomly at a frequency of 20 Hz. Each grating’s texture was shifted every 16.6 ms (i.e. each frame of the monitor with 60 Hz refresh rate) to generate apparent motion. Visual stimuli were presented at four possible locations that were equidistant from a central fixation spot. They were framed by a grey aperture (thickness: 0.15°, luminance: 110 cd/m^2^) of 8.97° × 14.15° in diameter to aid binocular fusion. Mask and target screen allocation (right, left eye) alternated between eyes across trials, to enhance suppression.

Auditory stimuli were 50 ms bursts of white noise. They were presented via six external speakers, placed above and below the monitor at 64 dB sound pressure level. Upper and lower speakers were aligned vertically and located centrally, 3° to the left and 3° to the right of the monitor’s centre (i.e. aligned with the flash location along the azimuth).

Psychophysical stimuli were generated and presented on a PC running Windows XP using the Psychtoolbox version 3.0.11^32^ running on MATLAB R2014a (Mathworks, Natick, Massachusetts). Staircase procedures were implemented using Palamedes toolbox^33^.

Visual stimuli were presented dichoptically using a gamma-corrected 30″ LCD monitor with a resolution of 2560 × 1600 pixels at a frame rate of 60 Hz (NVIDIA Quadro 600 graphics card). Auditory stimuli were digitized at a sampling rate of 44.8 kHz via an M-Audio Delta 1010LT sound card. Exact audiovisual onset timing was confirmed by recording visual and auditory signals concurrently with a photo-diode and a microphone.

### Experiment 1: Design

**Figure 1:**
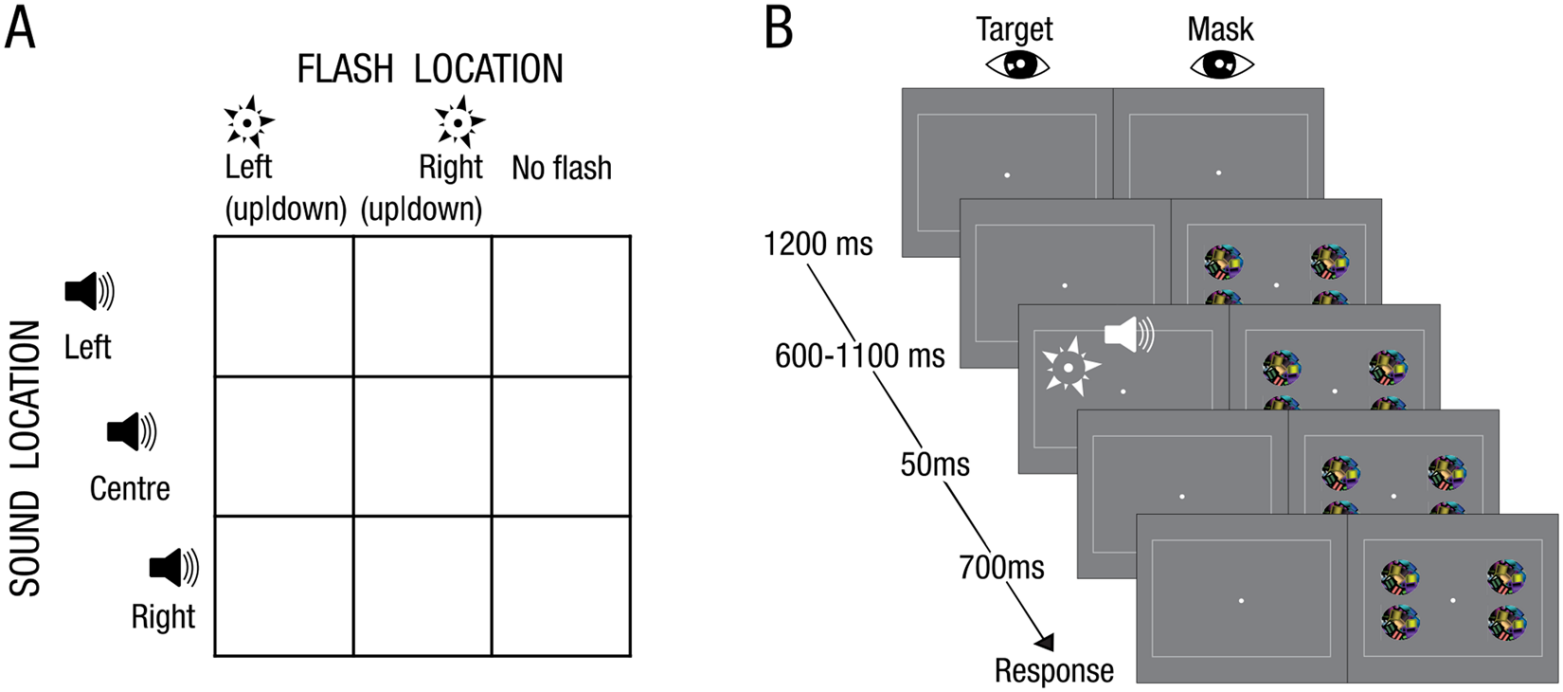
Experimental paradigm and procedure. (**A**) Experimental design: 3 × 3 factorial design with the factors: (1) Flash location: left (up|down), right (up|down), no flash; (2) Sound location: left, centre, right. The trials were categorized according to participants’ subjective visibility: Clear Image, Almost Clear Image, Weak Glimpse, Not Seen. (**B**) Example trial and procedure of dynamic flash suppression.

In a spatial ventriloquist paradigm, participants were presented with an auditory burst of white noise emanating from one of three potential locations: left, centre or right. In synchrony with the sound, one eye was presented with (i) no flash or a brief flash in participants’ (ii) left or (iii) right hemifield under dynamic continuous flash suppression to the other eye^30^. Hence, the 3 × 3 factorial design manipulated (1) ‘flash’ (3 levels: left flash, right flash, no flash) and (2) ‘sound location’ (3 levels: left sound, central sound and right sound) (Fig. 1A). In order to enable a flash localization task that is orthogonal to the sound localization, the flash could be presented either in the upper or lower hemifield (i.e. ±1.2° elevation from a grey central fixation dot). Hence, the flash was presented in the upper left quadrant, lower left quadrant, upper right quadrant or lower right quadrant (n.b. visual localization is highly precise close to the fixation point and has been shown to be equivalent for spatial discrimination along elevation and azimuth^34^).

Each trial started with the presentation of the fixation dot for duration of 1200 ms (Fig. 1B). Next, participants were presented with dynamic Mondrians to one eye that suppressed their awareness of signals presented to the other eye (dynamic continuous flash suppression). After a random interval of 600–1100 ms, a sound was played from one of three potential locations. On the flash present trials, a white disc was presented in one of the four quadrants for 50 ms in synchrony with the sound. The Mondrian masks were presented on the screen until participants had responded to all questions.

On each trial, participants responded to three questions in a self-paced manner within a total response window of 5 s: First, they reported the location of the beep (left, centre, right) via a three choice key press. Second, they rated the visibility of the flash (clear image, almost clear image, weak glimpse, not seen) according to a previously published Perceptual Awareness Scale^35,36^ (PAS) via a four choice key press. This Perceptual Awareness Scale encouraged participants to categorize trials as invisible, only if they were ‘completely invisible’. Third, they reported the location of the flash (upper or lower hemifield) via a two choice key press. Critically, we designed orthogonal auditory and visual tasks to minimize decisional biases between visual and auditory localization responses. In order to minimize response interference between responding to the set of three questions, we ensured that the responses mapped to distinct sets of buttons (i.e. 9 different buttons in total). The button/hand assignment and order of questions was counterbalanced across participants (for detailed keyboard mapping please see Fig. 1 in Supplementary Material).

This visibility judgment provided a subjective awareness criterion. Critically, prior to the main experiment we adjusted the flash’s luminance in adaptive staircases individually for each participant, such that the flash was visible only on 40% of the trials. This allowed us to quantify multisensory interactions as indexed by spatial ventriloquism (i.e. audiovisual spatial bias) for flashes that were visible (i.e. pooled over clear image, almost clear image, weak glimpse) or invisible (i.e. subjective awareness criterion^37^). Further, we could assess the information that is available for visual spatial localization during invisible trials and select participants that were not better than chance when locating flashes that they judged as invisible (i.e. the so-called chance performers). The latter allowed us to investigate the influence of flashes on sound localization, when they were invisible and unaware in a so-called objective sense (i.e. objective awareness criterion^37^).

Prior to the main experiment, participants were familiarized with stimuli and task. In particular, we adjusted the flash luminance in adaptive staircases (step size up: 8.8 cd/m^2^, step size down: 13.2 cd/m^2^), such that the flash was visible on 40% of the trials. The adaptive staircases were applied using a slightly modified experimental paradigm where the sound was presented always from the middle, the flash in one of the four quadrants and participants reported only flash visibility (yes, no) and location (up, down). After an initial long staircase (min 200 trials), we performed four times two interleaved adaptive staircases (convergence criterion: 8 reversals within last 10 trials).

During the main experiment participants completed a total of 8 experimental sessions, resulting in a total of 432 trials (i.e. 64 trials for each flash present condition and 16 trials for each flash absent condition). To maintain the targeted proportion of invisible trials (i.e. 40% visible trials), a staircase procedure was also used throughout the main experiment. To minimize the variability of the flash luminance during the main experiment we adjusted brightness of the flash in smaller step sizes (3.3 cd/m^2^) and only after 4 consecutive ‘not seen’ responses or after 3 consecutive ‘seen’ (including all three “partially visible” levels: clear, almost clear & weak glimpse) responses.

### Design limitations of Experiment 1 and motivation for Experiment 2

In the first study flash luminance was adjusted throughout the experiment to maintain a visibility level of approximately 40% (i.e. 60% of the trials were judged as invisible based on the four level Perceptual Awareness Scale^35,36^). This approach is ideal to ensure an approximate visibility level of 40% across all participants. However, it raises the possibility that the ventriloquist effect may be driven by flash stimuli with higher luminance values.

In the second study, we therefore adjusted the flash luminance only during the initial staircases individually for each subject, but held it constant throughout the entire main experiment. This experimental choice ensured that we compare the effect of physically identical flashes that were judged as visible or invisible on sound perception. Yet, because the subjective flash visibility fluctuates throughout the main experiment, this experimental choice induces significant variability in number of invisible and visible trials across participants. To ensure comparable reliability of parameter estimates across participants, we excluded subjects with insufficient number of trials (see exclusion criteria below). Yet, we note that the results were basically equivalent if all participants were included.

### Experiment 2: Design

The second study was identical to the first except that the flash luminance was adjusted only prior to the main experiment for each participant, but kept constant throughout the main experiment.

### Analysis for experiments 1 and 2

For data analysis, we reduced the four visibility levels to two visibility levels: 1. Visible = clear image + almost clear image + weak glimpse and 2. Invisible = not seen. Further, we pooled over flashes in upper and lower fields given their negligible elevation (i.e. ±1.2° visual angle). Unless otherwise stated, statistical analysis was identical for the two experiments.

For each participant, we coded their sound location responses as −1 for left, 0 for centre and 1 for right across trials. We estimated the *perceived sound location* for each of the 2 (flash location: left, right) × 3 (sound location: left, centre, right) conditions by averaging the localization responses across trials. Next, we averaged the perceived sound locations separately for trials where the flash was presented on the left and right and computed the difference in average perceived sound location for ‘visual right’ minus ‘visual left’ trials as the index for the spatial ventriloquist effect (for illustration please see Fig. 2 in Supplementary Material). A positive value of this index indicates that subject’s perceived sound location shifted towards the visual stimulus location (i.e. ‘attraction’) and negative value indicates that it is shifted away from the visual stimulus location (i.e. repulsion). A ventriloquist effect of zero means that participants were not influenced consistently across trials by the location of the flash.

This difference in perceived sound location, i.e. the ventriloquist effect, was then used as the dependent variable for all subsequent analyses. If the visual signal location attracts the perceived sound location, we would expect the difference to be significantly greater than zero. Given our a priori directed hypothesis all p-values are reported for right-tailed one sample t-tests.

The data analysis was performed in MATLAB (Mathworks, Natick, Massachusetts) with exception of unidirectional Bayes factors, which were computed using JASP^38^.

#### Exclusion criteria

For the reported results, we limited the analysis to subjects based on the following two exclusion criteria: First, we included only subjects that provided reliable visibility judgments as indicated by ‘better than chance localization accuracy’ (based on binomial test) for visible flashes (i.e. exclusion of six subjects from experiment 1 and three subjects from experiment 2). Second, to ensure reliable parameter estimation, we included only those participants who had at least 10 trials in each of the 2 (flash location: left vs. right) × 3 (sound location: left, middle, right) conditions, for both visible and invisible categories respectively (i.e. exclusion of three subjects from experiment 1 and six subjects from experiment 2). For individual distribution of PAS ratings see Fig. 3 in Supplementary Material. These exclusion criteria ensured that the computation of the ventriloquist effect was based on at least 60 trials. Yet, the minimal number of trials was even higher and amounted to 106 trials.

Further, we would like to emphasize that the results were basically equivalent when including all participants (apart from one for whom the ventriloquist effect could not be computed for invisible trials because only two trials were categorized as invisible and they did not fall into the corresponding conditions). In other words, significant results were again significant, non-significant results again non-significant, when no participants were excluded.

#### Direct comparison of spatial ventriloquism for visible and invisible trials

We investigated whether the audiovisual spatial bias (i.e. ventriloquist effect) was significantly different for visible (i.e. clear image, almost clear image and weak glimpse) and invisible trials using paired t-tests.

#### Spatial ventriloquism for visible and invisible trials

We investigated whether the ventriloquist effect was present independently for both invisible and visible flashes. Hence, we computed the ventriloquist effect separately for trials where visual signals were judged visible or invisible and tested whether the ventriloquist effect was significantly greater than zero in right-tailed one sample t-tests independently for visible and invisible trials. Demonstrating a ventriloquist effect for invisible trials suggests that flashes can influence perceived sound location, when participants are subjectively not aware of them (i.e. subjective awareness criterion^37^). Further, we investigated whether participants at the group level were at chance when locating a flash they judged invisible by comparing their accuracy scores against 50% chance performance in a right tailed one-sample t-test. For p values greater than 0.05 we computed Bayes factors to provide further evidence for the null hypothesis (i.e. 50% chance performance).

#### Spatial ventriloquism for invisible trials in chance performers

We asked whether invisible flashes are able to influence the perceived sound location, even in participants that are not better than chance when locating flashes they judged as invisible (i.e. objective awareness criterion^37^). Using directionally informed tests in i. classical statistics and ii. Bayesian inference we identified chance performers based on a binomial test on their flash localization performance on trials in which the flash was judged as invisible. First, to link with previous reports in the literature we defined chance performers individually based on a ‘null-result’ using a directional binomial test (i.e. ‘not significantly better than chance’) in classical statistics^39^. Second, as a ‘null-result’ in classical statistics is not decisive, we also used Bayesian statistics that allows one to quantify and compare the evidence for the null model that embodies the null-hypothesis in relation to an alternative model. Hence, using Bayes factors we compared a binomial distribution model that a priori fixes the probability to 0.5 (i.e. null model of chance performance) with one that includes the probability parameter p as a free parameter constrained by a positive prior distribution (i.e. directional binomial test). Please note that imposing a positive prior distribution makes the Bayesian test a more stringent for defining chance performers.

Nevertheless, because both selection criteria were applied in a non-crossvalidated fashion as is currently common in the field^3,40–42^, the definition of so-called chance performers can at least in part be susceptible to noise (inter-trial variability). As has been explained in detail in Shanks^43^, the flash localization accuracy may be lower in chance than non-chance performers in this experimental session because of performance noise and hence if measured again may increase, a statistical phenomenon referred to as ‘regression towards the mean’. Conversely, participants’ sound localization performance may be affected by -partly - independent noise. As a result, we may underestimate and falsely define observers as chance performers and conversely overestimate the ‘unaware’ ventriloquist effect.

#### Influence of question order on flash localization accuracy and ventriloquism

As described earlier we counterbalanced the task order (either: flash location – visibility – sound location or: sound location – visibility – flash location) across participants, because the task order can affect our analysis results in three important ways:

First, the presentation order of the questions can influence observer’s performance accuracy on the different tasks. For instance, as a result of memory noise, observers’ flash localization accuracy may be reduced when the flash localization task was presented last with additional consequences on the size of the ventriloquist effect. We therefore compared flash localization accuracy and the size of spatial ventriloquism for first vs. last task in a two-sample t-test.

Second, as a result of the reduced flash localization accuracy, we may have classified participants as chance performers mainly when the flash localization task was presented last. To assess the effect of task order on the classification as chance performers we compared the number of chance performers across the groups where the flash localization task was presented first vs. last in a Chi-square test.

Third, to assess the effect of task order on the exclusion of participants we compared the number of excluded participants across the groups where the flash localization task was presented first vs. last in a Chi-square test.

## Results (Experiments 1 and 2)

### Direct comparison of spatial ventriloquism for visible and invisible trials

A paired t-test comparing the ventriloquist effect for invisible and visible trials demonstrated a significantly greater ventriloquist effect for visible than invisible flashes (experiment 1: all participants: mean difference ± SEM = 0.44 ± 0.06, t(32) = 7.99, p < 0.001 chance performers: 0.47 ± 0.05, t(27) = 8.57, p < 0.001 experiment 2: all participants: 0.46 ± 0.07, t(17) = 6.45, p < 0.001; chance performers: 0.46 ± 0.07, t(12) = 6.34, p < 0.001).

### Spatial ventriloquism for visible and invisible trials

To investigate whether the ventriloquist effect emerged for visible and invisible flashes we performed one sample t-tests independently for each of these two visibility levels.

For visible trials, we observed a significant ventriloquist effect (i.e. audiovisual spatial bias) as expected based on numerous previous studies^28,29^ (experiment 1: mean ± SEM = 0.52 ± 0.06, right-tailed t(32) = 8.62, p < 0.001; Cohen’s d ± 95% Confidence Interval: 1.5 ± 0.55; experiment 2: 0.51 ± 0.06, t(17) = 9.14, p < 0.001; Cohen’s d: 2.15 ± 0.82).

Crucially, we also observed a significant ventriloquist effect for flash stimuli that participants judged as invisible (experiment 1: 0.08 ± 0.02, t(32) = 3.86, p < 0.001; Cohen’s d: 0.67 ± 0.5; experiment 2: 0.09 ± 0.03, t(17) = 3.22, p = 0.003; Cohen’s d: 0.76 ± 0.68). Participants’ flash localization accuracy was slightly above chance (experiment 1: mean ± SEM = 51.3 ± 0.7%; right-tailed t-test against 50% chance performance: t(32) = 1.88, p = 0.034; corresponding BF_01_ = 1.72; experiment 2: 53.6 ± 2%, t(17) = 0.91, p = 0.047; BF_01_ = 1.69), therefore the results reported so far only provide evidence that flashes that participants consider invisible (i.e. subjective awareness criterion) are still able to elicit a robust ventriloquist effect.

### Spatial ventriloquism for invisible trials in chance performers defined based on classical statistics

To ensure that the ventriloquist effect for invisible trials was not driven by participants that had residual visual information for visual flash localization, we repeated this analysis using the more stringent so-called objective criterion for perceptual awareness. Hence, we included only those subjects that were individually not better than chance when locating an invisible flash based on binomial testing (i.e. objective awareness criterion). The constraint of individual chance performance reduced the number of subjects that could be included in the analysis (experiment 1: n = 28; experiment 2: n = 13).

**Figure 2:**
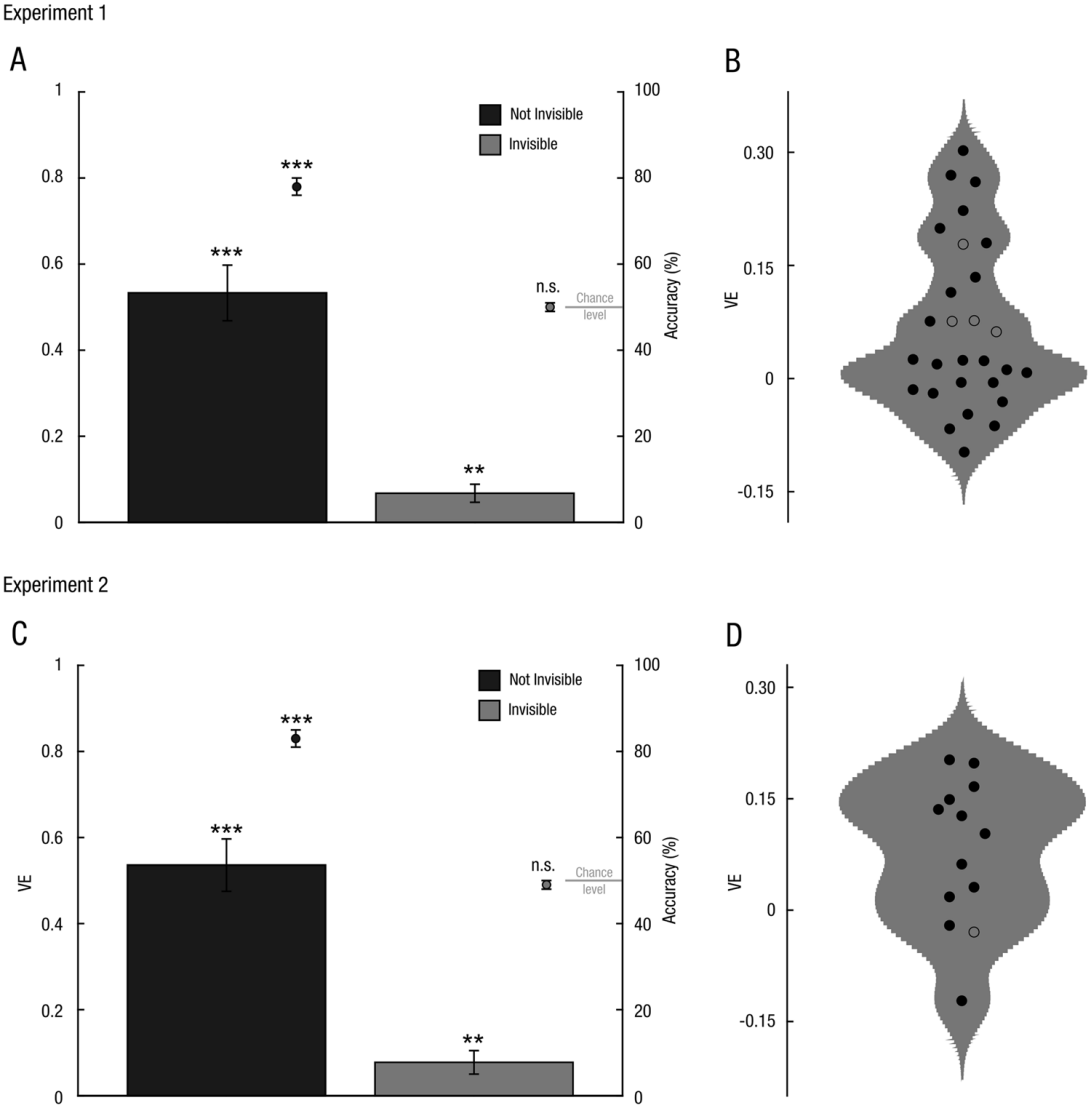
Results for chance performers: experiment 1 (n = 28) & experiment 2 (n = 13). (**A**,**C**) Bar plots showing the ventriloquist effect in chance performers (VE, across subjects mean ± SEM) for visible and invisible flashes (left axis). The VE was significantly greater than zero for both visible and invisible trials. The markers show the accuracy (across subjects mean ± SEM) for flash localization (right axis: percentage correct). (**B**,**D**) Violin plots showing the distribution of individual ventriloquist effects for invisible trials in chance performers identified based on classical and Bayesian binomial tests. All dots represent subjects with not significantly better than chance performance based on classical statistics. Filled dots show subjects, for which BF_01_ for Bayesian binomial test was also greater than 3 (i.e. positive evidence for the null model of chance performance). The mass of the probability distribution is clearly above zero. Markers show the individual data points. ***p < 0.001, **p < 0.01, n.s. p > 0.05.

Nevertheless, despite the reduced number of subjects, we still observed a highly significant ventriloquist effect for invisible trials (experiment 1: 0.07 ± 0.02, t(27) = 3.22, p = 0.002; Cohen’s d: 0.61 ± 0.54; experiment 2: 0.08 ± 0.03, t(12) = 2.84, p = 0.007; Cohen’s d: 0.79 ± 0.8) (Fig. 2).

In other words, both experiments jointly demonstrated that an invisible flash attracted the perceived sound location even in subjects that were not better than chance when locating the flash, they judged as invisible. The flash localization accuracy (i.e. across subjects mean) at the group level was not significantly better than chance but nearly equal to 50% (experiment 1: 50.3 ± 0.6%; t(27) = 0.41, p = 0.341; BF_01_ = 3.53; experiment 2: 49.5 ± 1.1%, t(12) = −0.5, p = 0.687; BF_01_ = 4.97) (Fig. 2A,C). Hence, when selecting participants based on the objective awareness criterion the Bayes factors at the group level provided strong evidence for the null-hypothesis, i.e. that participants were not better than chance when locating the flash, independently for each experiment.

### Spatial ventriloquism for invisible trials in chance performers individually defined based on Bayesian statistics

As classical statistics does not allow the acceptance of the null-hypothesis, we also used unidirectional Bayesian tests that can formally provide positive evidence for the null-hypothesis of chance performance individually for each participant. Specifically, we computed Bayes factors comparing the evidence for the null-model of chance performance with the evidence for alternative model of better than chance performance and selected only subjects as chance performers with Bayes factors >3 (i.e. positive evidence for the null-model). This reduced the number of included chance performers in both experiments (experiment 1: n = 24, experiment 2: n = 12, mean flash localization accuracy: 49.5 ± 0.6% and 48.9 ± 1% respectively).

Nevertheless, despite this more stringent objective awareness criterion we again observed significant ventriloquist effect for invisible trials (experiment 1: 0.06 ± 0.02, right-tailed t(23) = 2.61, p = 0.008; Cohen’s d: 0.53 ± 0.58; experiment 2: 0.09 ± 0.03, t(11) = 3.09, p = 0.005; Cohen’s d: 0.89 ± 0.84).

### Influence of question order on flash localization accuracy and ventriloquism

A two sample t-test did not reveal a significant effect of question order on flash localization accuracy (experiment 1: visible: p = 0.209, t = −1.28 invisible: p = 0.454, t = −0.76; experiment 2: visible: p = 0.822, t = 0.23, invisible: p = 0.176, t = −1.42) or the size of ventriloquist effect (experiment 1: visible: p = 0.703, t = −0.39, invisible: p = 0.141, t = −1.51; experiment 2: visible: p = 0.537, t = −0.63, invisible: p = 0.523, t = −0.65).

Likewise, a Chi-square test did not reveal a significant effect of question order on the number of subjects classified as chance performer using criteria based on binomial testing (experiment 1: χ^2^ = 0.50, p = 0.478; experiment 2: χ^2^ = 1.68, p = 0.196) or Bayesian statistics (experiment 1: χ^2^ = 2.25, p = 0.134; experiment 2: χ^2^ = 0.45, p = 0.502). Further, question order did not significantly affect the exclusion of participants (experiment 1: χ^2^ = 0.90, p = 0.343; experiment 2: χ^2^ = 0.37, p = 0.541).

## Discussion

Using continuous flash suppression and spatial ventriloquism we demonstrate that unconscious signals in the visual modality influence how humans construct their auditory perceptual world. In particular, we have shown that flashes judged as invisible alter the perceived location of concurrent sounds, even when participants are at chance when locating the flash. These results suggest that auditory and visual inputs are integrated into spatial representations at least to some extent in the absence of subjective and objective perceptual awareness.

Accumulating evidence has shown that audio-visual integration of speech information is abolished when visual facial movements are rendered unconscious via multistable perception, binocular rivalry or flash suppression^19,20^ highlighting the role of perceptual awareness in multisensory integration. This raises the question whether consciousness is a generic prerequisite for multisensory integration and is also required for or associated with interactions of spatial signals as indexed by the ventriloquist effect.

Our findings demonstrate that spatial ventriloquism is profoundly modulated by the visibility of the flash. While a strong ventriloquist effect was observed for visible trials, it was attenuated when the flash was judged as invisible. Nevertheless, a robust ventriloquist effect was observed across both experiments for trials when participants judged the flash as invisible (i.e. subjective awareness criterion).

Moreover, across both experiments the ventriloquist effect persisted even for invisible flashes when participants showed chance performance on flash localization (i.e. objective awareness criterion).

Collectively, our two experiments show that invisible flashes, that human observers are not aware of, can influence where they report sounds, that they are aware of.

Invisible flashes may influence sound localization during continuous flash suppression via at least three distinct neural circuitries. First, an invisible flash may interact with auditory signals via subcortical mechanisms such as the colliculo-pulvinar pathway^44,45^ that has previously been implicated in mediating activations along the dorsal stream into the intraparietal sulcus under CFS^46^, but see^47–49^. Because participants were engaged in a spatial localization task and the ventriloquist effect relies on integration of spatial representations from vision and audition, the dorsal stream may be critical in our paradigm^27,50,51^. Second, it may modulate sound processing via sparse direct connectivity between primary auditory and visual areas^24,52^. Third, some flash-induced neural activity may evade flash suppression and propagate across the cortical hierarchy into higher order association areas such as intraparietal sulcus or even prefrontal cortices^23–25,27,53–56^. While this activation may not be sufficient to allow better than chance flash location, it enables to bias participants’ sound localization.

The ventriloquist effect may be smaller for invisible than visible flashes, because invisible flashes may evoke weaker or less reliable activations than visible flashes already at the primary cortical level as a result of state-dependent effects or various sources of internal neural noise^57^. The level of neural activity then concurrently determines (i) whether the flash is able to enter perceptual awareness and (ii) the precision of the spatial representation and thereby the strength of the ventriloquist effect^58,59^. Thus, visible flashes would induce a ventriloquist effect via the same neural circuitries as invisible flashes and induce a greater ventriloquist effect, as they induce higher neural activity and thus more precise spatial representations in visual cortices.

Alternatively, visible flashes may induce a stronger ventriloquist effect by employing additional neural circuitries (e.g. via higher order association areas) that are not engaged by weaker invisible flashes. In this account the spatial representations elicited by a flash at the primary cortical level may be preserved, yet be less effective in influencing the sound processing system. This latter account dovetails nicely with current perspectives on the neural organization of multisensory integration. Specifically, auditory and visual information are thought to be integrated via multiple circuitries including subcortical mechanisms, direct connectivity between primary sensory areas and convergence in higher order association areas^25,27,54,55^. Moreover, it is well established that multisensory integration progressively increases along the cortical hierarchy with only about 15% neurons showing multisensory properties in primary sensory areas^22^ and more than 50% in classical association areas such as intraparietal or superior temporal sulci^53^.

Thus, when a visual flash escapes the continuous flash suppression and enters participants’ awareness, a strong ventriloquist effect emerges most likely via integration in association areas such as intraparietal sulci (IPS) that contain exuberant multisensory neurons and may potentially amplify multisensory integration via feed-back loops with lower level sensory areas. By contrast, when continuous flash suppression blocks neural activity at least to some extent from propagating into higher order association areas, audio-visual interactions are greatly attenuated or even abolished leading to a smaller ventriloquist effect. Under this ‘multiple neural circuitries’ account, auditory and visual signals interact most likely at both pre- and post-aware processing stages by placing different demands on distinct neural circuitries (e.g. direct connectivity vs. higher order association cortices).

The combination of different psychophysical blinding methods that affect visual processing at variable depths^59–61^ may enable us to better dissociate between these different mechanisms. For instance, while flash suppression is thought to affect processing in primary visual areas alike contrast modulation^59^, attentional blink may alter processing mainly at higher attentional levels. In fact, we suspect that our current paradigm potentially combines both mechanisms by placing attentional demands at four locations.

In conclusion, to our knowledge our findings provide the first demonstration that invisible flashes can alter and bias where we perceive sounds. These results suggest that low level sensory information can interact across sensory modalities at least to some extent prior to perceptual awareness. Nevertheless, audiovisual interactions as indexed by spatial ventriloquism were stronger for visible relative to invisible flashes that participants were not able to locate better than chance. This raises the possibility that aware visual signals may also engage multisensory mechanisms in higher order association areas or other neural circuitries that are less engaged in the absence of perceptual awareness. Future studies using EEG and fMRI are needed to identify the neural systems that enable audio-visual interactions in the presence and absence of subjective and objective awareness.

## Electronic supplementary material

Supplementary Material

## Data Availability

The datasets generated and analysed during the current study are available from the corresponding author upon request.
