# Supplementary material for: Invisible Flashes Alter Perceived Sound Location
Source: Sci Rep. 2018 Aug 17;8:12376. doi: 10.1038/s41598-018-30773-3 (PMC6098122; doi:10.1038/s41598-018-30773-3)
Supplement: Supplementary file 1 — Supplementary Material [file 41598_2018_30773_MOESM1_ESM.pdf]

INVISIBLE FLASHES ALTER PERCEIVED SOUND LOCATION

Patrycja Delong[1*], Máté Aller[1], Anette S. Giani[2], Tim Rohe[2], Verena Conrad[2,] Masataka Watanabe[2] & Uta Noppeney[1, 2]

[1] Computational Neuroscience and Cognitive Robotics Centre, University of Birmingham, B15 2TT Birmingham, UK
[2] Max Planck Institute for Biological Cybernetics, 72076 Tübingen, Germany

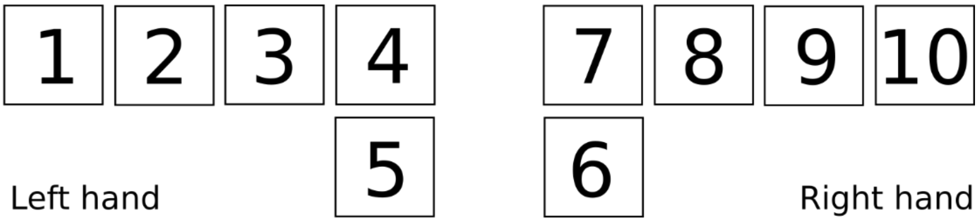

Supplementary Figure 1

Keyboard mappings. Participants used 9 different buttons to respond. The button/hand assignment and order of questions was counterbalanced across participants (i.e. subject reported visibility with left hand and flash/sound locations with right hand or vice versa). Version A: visibility (PAS scale) - buttons 1-4; sound location (left, center, right) - buttons 8-10; flash location (top, down) - buttons 6 & 7. Version B: visibility (PAS scale) - buttons 7-10; sound location (left, center, right) - buttons 1-3; flash location (top, down) - buttons 4 & 5.

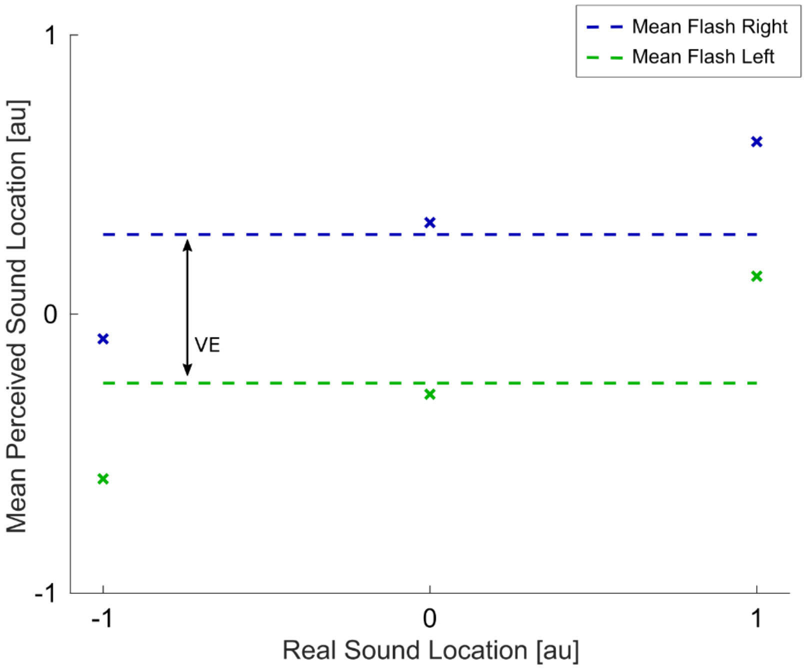

Supplementary Figure 2

Computation of Ventriloquist Effect. The crosses indicate the mean perceived sound location (in arbitrary units) for each of the 2 flash (left, right) x 3 sound (left, centre, right) conditions. Mean of the differences between 'visual right' (blue) and 'visual left' conditions serves as index of spatial ventriloquism. Results for visible condition for chance performers from experiment 1 were used for illustration.

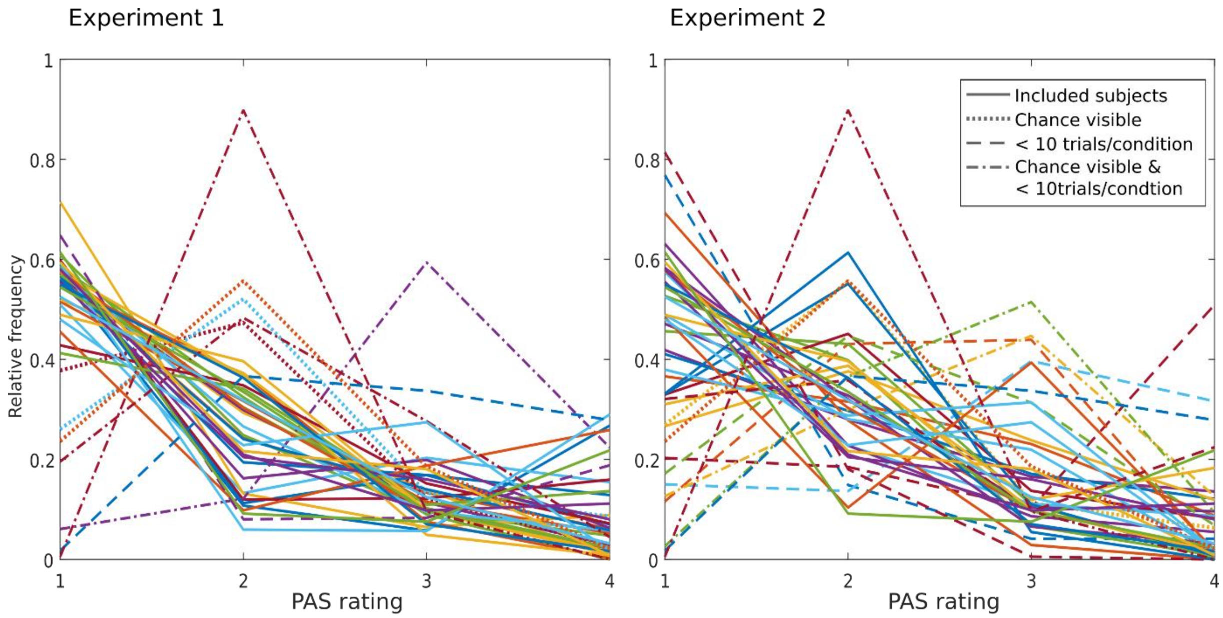

Supplementary Figure 3

Visibility judgement. Figures show proportions of perceptual awareness scale (PAS) ratings for subjects from experiment 1 and 2. Each line represents an individual participant. Solid = included in the analysis; dotted = excluded because of chance performance on flash localization for visible trials; dashed = excluded because less than 10 trials in each condition; dotted-dashed = excluded because of both, i.e. chance performance for flash localization accuracy for visible trials and less than less than 10 trials in each condition.
